# Impact of initial empirical antimicrobial choice and cause of in-hospital death in patients with nursing and healthcare-associated pneumonia (NHCAP): A retrospective study

**DOI:** 10.20407/fmj.2021-019

**Published:** 2022-01-25

**Authors:** Kenichi Kato, Kazunobu Kuwabara, Kiyotaka Ono, Yusuke Kito, Tatsuyoshi Yokoi, Takazumi Yoshida, Keisuke Kato, Masahiro Hirose, Daijo Inaguma, Takahiko Horiguchi

**Affiliations:** 1 Department of Internal Medicine/Respiratory Medicine, Fujita Health University, School of Medicine, Nagoya, Aichi, Japan; 2 Department of Internal Medicine, Fujita Health University Bantane Hospital, Nagoya, Aichi, Japan; 3 Toyota Regional Medical Center, Toyota, Aichi, Japan

**Keywords:** Nursing and healthcare-associated pneumonia, Pneumonia, Empiric antimicrobial therapy

## Abstract

**Objectives:**

To examine the differences in antimicrobial selection outcomes in nursing and healthcare-associated pneumonia (NHCAP) patients with and without risk factors for drug-resistant pathogen (RDRP) infection, and to identify the cause of in-hospital death after improvement of NHCAP.

**Methods:**

We conducted a retrospective analysis of the medical records of hospitalized adult patients with NHCAP. NHCAP patients were divided into the RDRP and non-RDRP groups. The RDRP group was further classified into the narrow and broad subgroups according to the type of empirical antimicrobial agent selected. The difference in mortality between these subgroups was then examined. The cause of all in-hospital deaths was also evaluated.

**Results:**

e evaluated 220 patients with NHCAP. There was no difference in mortality between the narrow and broad subgroups (11.8% vs. 15.4%, p=0.655). Among the group with improved NHCAP, 11.3% (n=23/203) died in hospital before discharge. Although the causes of death in patients who improved after NHCAP were diverse, the most common was recurrence of pneumonia.

**Conclusions:**

Empirical antimicrobial therapy for NHCAP may not always require selection of broad-spectrum antimicrobial agents, as has been previously reported. Patients with NHCAP may die from other causes, even after NHCAP has improved.

## Introduction

Pneumonia can be classified into community-acquired pneumonia (CAP) and hospital-acquired pneumonia (HAP) according to whether it occurs in the community or during hospitalization. This classification is important because there is a difference in the causative organism between CAP and HAP, which calls for different treatment strategies. The 2005 joint guidelines published by the American Thoracic Society (ATS) and the Infectious Diseases Society of America (IDSA) included a new category of pneumonia, that is, healthcare-associated pneumonia (HCAP).^1^ HCAP is a type of pneumonia that falls between CAP and HAP and is associated with a higher frequency of inappropriate initial empirical antibiotic therapy, as well as a higher mortality rate than CAP.^2–5^

Similar to the USA, Japan’s population is aging, and an increasing number of patients are being admitted to nursing care facilities. However, owing to differences in the insurance system in Japan, many long-term care facilities that are considered nursing homes in the USA are classed as hospitals in Japan. Furthermore, many cases of pneumonia that are considered HCAP in the USA are treated as HAP in Japan. Therefore, in 2011, the Japan Respiratory Society (JRS) Guidelines proposed a new category, nursing- and healthcare-associated pneumonia (NHCAP), which better reflects the healthcare insurance system, including long-term care insurance, as well as the patterns of drug-resistant pathogens.^6^ According to these guidelines, antimicrobial agents that cover *Pseudomonas aeruginosa* and methicillin-resistant *Staphylococcus aureus* (MRSA), such as tazobactam/piperacillin (TAZ/PIPC), carbapenems, and vancomycin, are recommended for NHCAP in individuals at increased risk for developing drug-resistant infections because of their background.

It is noteworthy that, in actual clinical practice, even if a patient is at risk of developing a drug-resistant bacterial infection, the attending physician may choose a narrow-spectrum antimicrobial agent as an empirical choice. Several studies have shown that the choice of broad-spectrum antimicrobial agents does not necessarily improve the outcome of NHCAP.^7,8^ However, the impact of empirical antimicrobial selection on NHCAP remains unclear. In addition, because NHCAP mainly occurs in older patients, even if their pneumonia at admission improves, they may have a pneumonia flare-up or die from other unrelated complications before discharge. Despite this situation, which is important for patients and their families to know, no previous studies have examined the outcomes in patients admitted with NHCAP from the time of improvement of NHCAP to discharge from hospital.

This retrospective study compared differences in outcomes according to choice of initial antimicrobial agents for patients with NHCAP with risk factors for drug-resistant bacterial infection according to the Japanese guidelines, and to further identify in-hospital deaths after improvement of NHCAP at our hospital.

## Methods

### Patients and study design

This was a retrospective study of adult patients hospitalized with NHCAP between April 2015 and March 2018 at the Fujita Health University Bantane Hospital (a 370-bed university teaching hospital in Nagoya, Aichi, Japan). Patients with acute interstitial pneumonia or pulmonary tuberculosis were excluded. The patients were categorized into those with risk factors for infection with drug-resistant pathogens (RDRP group) and those without risk factors for infection with drug-resistant pathogens (non-RDRP group). The RDRP group was further classified into the narrow-spectrum antimicrobial subgroup (narrow subgroup) and broad-spectrum antimicrobial subgroup (broad subgroup) according to the type of antimicrobial agent empirically selected.

All clinical data, including age, sex, A-DROP score,^6^ use of antibacterial agents, microbiological examinations, and clinical outcomes up to the time of death or discharge from hospital, were retrospectively collected from medical records.

This study was approved by the Medical Research Ethics Committee of Fujita Health University (approval no. HM18-146). The study conformed to the principles outlined in the Declaration of Helsinki, and the need for informed consent was waived due to the retrospective nature of the study.

### Definition

Pneumonia was defined as radiographic evidence of an infiltrate with at least one of the following symptoms: fever, cough, sputum production, dyspnea, or pleuritic pain. NHCAP and risk factors for infection with drug-resistant pathogens were defined according to the JRS guidelines.^6^ NHCAP was defined as meeting any of the following criteria: 1) residence in a long-term nursing or healthcare home; 2) discharge from hospital in the preceding 90 days; 3) older or physically disabled persons who needed healthcare; and 4) continuous endovascular therapy in an ambulatory setting (including dialysis, antibiotics, anticancer drugs, and immunosuppressants).

Patients who met any of the following criteria were classified into the RDRP group: 1) received antibiotics in the past 90 days; 2) current tube feeding; and 3) history of MRSA isolation. If none of these factors were met, the patients were classified into the non-RDRP group. The A-DROP scoring system assessed the following parameters: 1) age ≥70, ≥75 years; 2) dehydration (blood urea nitrogen [BUN] ≥21 mg/dL); 3) respiratory failure (SaO_2_ ≤90% or PaO_2_ ≤60 mmHg); 4) orientation disturbance (confusion); and 5) low blood pressure (systolic blood pressure ≤90 mmHg).

Orientation disturbance was defined as the equivalent of a Glasgow Coma Scale score <15. An A-DROP score ≥3 points was defined as severe pneumonia.^6^ A positive result on the sputum bacterial culture was defined as more than a third of the bacterial area of the Petri dish. Narrow-spectrum antimicrobials were defined as those that did not cover *P. aeruginosa* or MRSA (e.g., ampicillin, sulbactam/ampicillin (SBT/ABPC), and first-, second-, or third-generation cephalosporins). Broad-spectrum antimicrobials were defined as those covering *P. aeruginosa* or MRSA, such as TAZ/PIPC, fluoroquinolone, carbapenem, and anti-MRSA drugs (such as vancomycin). Escalation was defined as a change from an empirically selected antimicrobial agent to an antimicrobial agent with a broader spectrum. NHCAP-related deaths were defined by respiratory physicians based on death occurring up to day 14 of hospitalization. The cause of in-hospital death after improvement of NHCAP was determined from the patients’ medical records by the respiratory physician.

### Statistical analysis

Data are presented as the frequency and percentage or the mean and standard deviation unless otherwise indicated. The medians of the two independent groups were compared using the Mann–Whitney *U* test. The means of the two independent groups were compared using Student’s *t* test. The chi-square test was used to compare categorical data. Kaplan–Meier curves were used to estimate the cumulative survival ratio. All statistical analyses were performed using StatMate version 3.19 (ATMS Co. Ltd., Tokyo, Japan) and R version 4.0.5 (R Foundation for Statistical Computing). P<0.05 was considered statistically significant.

## Results

### Patient characteristics

**Figure 1:**
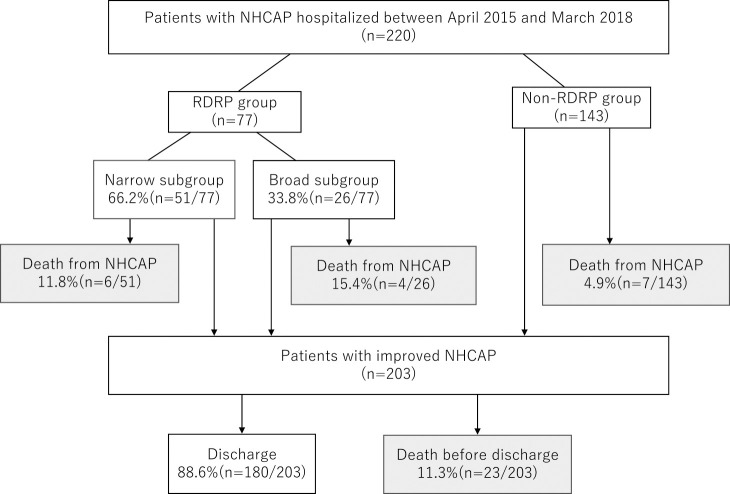
Flowchart showing the participant selection process. RDRP group, patients with risk factors for drug-resistant pathogen infection; non-RDRP, patients without risk factors for drug-resistant pathogen infection; narrow subgroup, patients treated with narrow-spectrum antimicrobials for NHCAP; broad subgroup, patients treated with broad-spectrum antimicrobials for NHCAP. NHCAP, nursing and healthcare-associated pneumonia.

We evaluated 220 patients with NHCAP during the study period: 35.0% (n=77) were categorized in the RDRP group and 65.0% (n=143) in the non-RDRP group (Figure 1). In the RDRP group, 66.2% (n=51) patients were in the narrow subgroup and 33.8% (n=26) in the broad subgroup. The baseline characteristics of the patients are shown in Table 1.

There was no significant subgroup difference in the frequency of underlying diseases, severe pneumonia (A-DROP >3), or frequency of sputum culture isolation of *P. aeruginosa* (11.8% vs. 19.2%, p=0.493) and MRSA (9.8% vs. 11.5%, p=1.000). All empirical antimicrobial therapy was monotherapy, and SBT/ABPC were the most commonly selected narrow-spectrum antimicrobials (90%, n=46), and TAZ/PIPC were the most commonly selected for broad-spectrum antimicrobial therapy (81%, n=21). The rate of change to broader-spectrum antimicrobial agents was higher in the narrow than in the broad subgroup (35.2% vs. 11.5%, p=0.032). The duration of antimicrobial administration was shorter in the narrow subgroup (8.8±3.3 days vs. 9.5±4.3 days, p=0.378; Table 2). None of the differences was significant.

In the narrow subgroup (n=51), 65% (n=33) of patients had improved NHCAP without escalation (success group), and 35% (n=18) required escalation after initial empirical narrow-spectrum antimicrobial therapy (failure group). Escalation was required in all six fatal cases. There were no significant differences in any factors between the success and failure groups (Table 3).

### Death from NHCAP

The NHCAP-related mortality rate was 7.7% (n=17/220), and the mortality rate was significantly higher in the RDRP group than in the non-RDRP group (13% vs. 4.9%, p=0.032). In the RDRP group, *P. aeruginosa* was identified in the sputum in 10% (n=1/10) and MRSA in 20% (n=2/10) of fatal cases caused by NHCAP. In the non-RDRP group, *P. aeruginosa* was identified in the sputum in none and MRSA in 14.3% (n=1/7) of fatal cases caused by NHCAP. There was no difference in mortality between the narrow and broad subgroups (11.8% vs. 15.4%, p=0.655) (Figure 1, Table 2). The median time from admission to death from NHCAP was 7 (interquartile range [IQR], 5.3–8.6) and 5 (3–7) days in the RDRP and non-RDRP groups, respectively. There was no significant difference in the duration from admission to death from NHCAP between the narrow and broad subgroups (8 [6.3–9.9] vs. 6 [4.5–7.3] days, p=0.170).

### In-hospital deaths after NHCAP improvement

Forty of 220 (18.2%) NHCAP patients died in hospital from any cause. There was no significant difference in underlying disease between patients who were discharged from hospital and patients who died in hospital from any cause.

**Figure 2:**
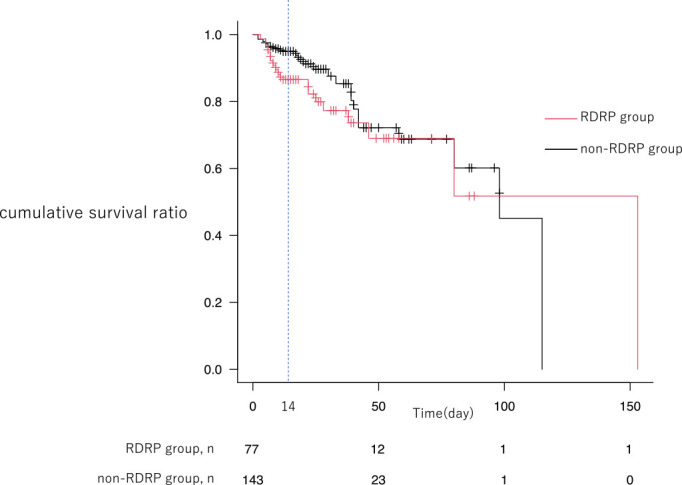
Kaplan–Meier cumulative survival curves for all patients. “death” as event and “discharge” as censored. RDRP group, patients with risk factors for drug-resistant pathogen infection; non-RDRP group, patients without risk factors for drug-resistant pathogen infection.

Among the group with improved NHCAP, 11.3% (n=23/203) died in hospital before discharge (Figure 2), and there was no significant difference between the RDRP and non-RDRP groups (11.9% vs. 11.0%, p=0.818). The median time from improvement of NHCAP to death was 22 (IQR, 14.5–34.6) days, with no significant difference between the RDRP and non-RDRP groups: 19.5 (16.3–31.2) vs. 30 (12.5–38) days (p=0.6427).

**Figure 3:**
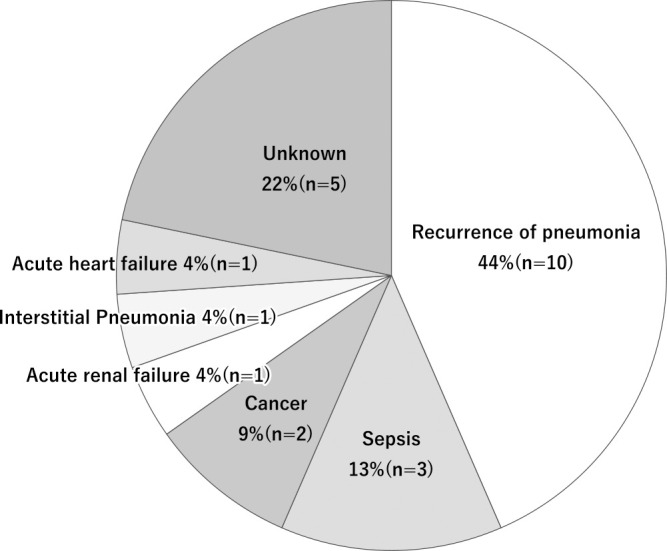
Causes of in-hospital death in patients who improved after NHCAP. NHCAP, nursing and healthcare-associated pneumonia.

The causes of in-hospital death in patients who improved after NHCAP were recurrence of pneumonia, 45% (n=10); sepsis, 14% (n=3); cancer, 9% (n=2); acute renal failure, 4% (n=1); interstitial pneumonia, 5% (n=1); acute heart failure, 5% (n=1); and unknown, 24% (n=5) (Figure 3). All of the unknown cases were unexpected and sudden deaths.

## Discussion

Data on the outcomes of patients admitted with NHCAP from the time of improvement to discharge are limited. This retrospective study found that there were no differences in the severity of pneumonia and in mortality of NHCAP between the narrow and broad subgroups within the RDRP group.

A previous retrospective study of NHCAP found that the mortality rate was not significantly different between patients receiving broad-spectrum or narrow-spectrum antimicrobial agents.^7^ However, another study found a higher mortality rate in patients administered broad-spectrum antimicrobial agents.^8^ Komiya et al. reported that aspiration pneumonia was independently associated with an increase in 30-day mortality after adjusting for other variables, including pneumonia, performance status, severity, and treatment failure because of antimicrobial-resistant infections.^9^ It is important to note that the concept of NHCAP is similar to HCAP. A cohort study in the UK concluded that failure to follow the current HCAP guidelines had no adverse effect on the ultimate outcome, and strategies for the empirical management of HCAP should be tailored to the local epidemiological context.^10^ According to a previous study on HCAP and HAP, adherence of empirical treatment to ATS/IDSA guidelines was associated with increased mortality.^11^ The authors questioned the need for concomitant empirical therapy for Gram-negative bacterial infection, even in patients who are clearly at high risk for multidrug-resistant infection.^11^

The above reports indicate the need to consider other factors apart from empirical antimicrobial selection, such as the patient’s underlying disease state, including contact dysphagia, and activities of daily living. In the present study, among the patients with improved NHCAP, 11.3% (n=23/203) died in hospital before discharge. Although the median duration of hospitalization for all patients was 20 (IQR, 11.8–29.8) days, we were unable to confirm death after discharge. In the Kaplan–Meier curve, the mortality rate up to ~14 days following hospitalization was higher in the RDRP group than in the non-RDRP group. However, 20–30 days after improvement of NHCAP, the mortality rates from other causes of the two groups were similar. The causes of death after improvement of NHCAP were diverse, although recurrence of pneumonia was the most common. Although we did not follow up patients after they were discharged from our hospital, we showed that patients hospitalized with NHCAP were at risk of death for various reasons, even after NHCAP improved.

The limitations of our study were its retrospective nature, and the fact that there was no set protocol for the choice of initial empirical antimicrobial agents; therefore, the choice of antimicrobial agents was left to the clinician. Hence, empirical antimicrobial agents may have been selected based on factors not considered in this study, such as performance status. In addition, there were many cases of spectrum expansion (escalation) after treatment with an empirical antimicrobial agent, which may have affected the patients’ outcome. These limitations mean that the results of this study do not establish the appropriate initial empirical antimicrobial agents for NHCAP. Therefore, prospective randomized trials are required to clarify the impact of empirical antimicrobial selection on NHCAP outcomes.

## Conclusions

There were no differences in the severity of pneumonia and mortality of NHCAP between the narrow and broad subgroups within the RDRP group. The frequency of all deaths from NHCAP was 7.7% (n=17/220). Among patients with improved NHCAP, 11.3% (n=23/203) died in hospital before discharge.

## Figures and Tables

**Table1:** Patients’ baseline characteristics

	RDRP group (n=77)^a^			non-RDRP group (n=143)^b^
	Narrow subgroup^c^ (n=51)	Broad subgroup^d^ (n=26)	p	
Sex (male)	55% (28)	50% (13)	0.684	58% (83)
Age (y)	83.0±12.9	80.5±9.4	0.092	86.0±7.3
Underlying disease				
COPD	16% (8)	19% (5)	0.752	13% (18)
Chronic heart failure	37% (19)	35% (9)	1.000	34% (49)
Chronic renal failure	12% (6)	23% (6)	0.205	20% (28)
Diabetes	16% (8)	12% (3)	0.741	15% (21)
Cerebrovascular disease	41% (21)	31% (8)	0.459	29% (41)
Parkinson disease	18% (9)	8% (2)	0.316	9% (13)
Dementia	47% (24)	38% (10)	0.628	55% (79)
Cancer	8% (4)	15% (4)	0.432	4% (6)
Transported by ambulance	39% (20)	54% (14)	0.221	48.2% (69)
Days of admission, median, IQR	22 (11–42.5)	23.5 (12.25–34.75)	0.605	19 (12–36.5)
A-DROP score ≥3	47% (24)	50% (13)	0.807	53.8% (77)
Older age^e^	86% (44)	88% (23)	1.000	97% (139)
BUN ≥21 mg/dL	41% (21)	31% (8)	0.459	49.6% (71)
Respiratory failure^f^	43% (22)	61% (16)	0.127	48.2% (69)
Orientation disturbance^g^	57% (29)	50% (13)	0.5673	55.2% (79)
Low blood pressure^h^	8% (4)	8% (2)	1.000	3.5% (5)
Systolic blood pressure (mmHg)	127.7±27.2	127.3±25.3	0.953	134.8±26.2
Diastolic blood pressure (mmHg)	72.8±21.4	72.2±18.0	0.914	75.0±16.5
Heart rate (bpm)	92.8±22.6	100.0±18.8	0.167	91.7±19.5
BUN (mg/dL)	24.3±17.5	21.9±19.8	0.589	26.8±20.9
WBC (/μL)	10 507±4682	10 680±5090	0.882	10 693±4472
Hb (g/dL)	11.5±2.1	11.0±1.8	0.301	11.4±2.0
Ht (%)	35.3±5.9	33.7±5.9	0.277	34.7±6.1
Platelet (×10^4^/μL)	25.2±91.1	27.4±12.7	0.391	21.3±7.3
CRP (mg/dL)	10.2	12.2	0.353	9.9±7.8
Alb (g/dL)	2.99±0.68	2.83±0.49	0.323	3.05±0.56
Na (mEq/L)	137.4±6.9	135.2±5.6	0.160	137.3±5.5
Sputum culture results				
* Pseudomonas aeruginosa*	11.8% (6)	19.2% (5)	0.493	4.2% (6)
MRSA	9.8 (5)	11.5 (3)	1.000	5.6% (8)
empiric antimicrobial				
Narrow spectrum				
SBT/ABPC	90% (46)	0		64% (92)
3^rd^ cefems	10% (5)	0		19% (27)
Broad spectrum				
TAZ/PIPC	0	81% (21)		12% (17)
Carbapenems	0	12% (3)		3% (4)
Quinolones	0	4% (1)		2% (3)
4^th^ cefems	0	4% (1)		1% (2)
Anti-MRSA	0	0% (0)		0% (0)

^a^ Patients with risk factors for drug-resistant pathogen infection. ^b^ Patients without risk factors for drug-resistant pathogen infection. ^c^ Patients treated with narrow-spectrum antimicrobials for NHCAP. ^d^ Patients treated with broad-spectrum antimicrobials for NHCAP. ^e^ ≥70 years for males and ≥75 years for females. ^f^ SaO_2_ ≤90% or PaO_2_ ≤60 mmHg. ^g^ Glasgow Coma Scale score <15. ^h^ Systolic blood pressure ≤90 mmHg. Alb, albumin; BUN, blood urea nitrogen; COPD, chronic obstructive pulmonary disease; CRP, C-reactive protein; Hb, hemoglobin; Ht, hematocrit; MRSA, methicillin-resistant *Staphylococcus aureus*; NHCAP, nursing and healthcare-associated pneumonia; SBT/ABPC, sulbactam/ampicillin; TAZ/PIPC, tazobactam/piperacillin; WBC, white blood cell.

**Table2:** Characteristics of patients with in-hospital mortality

	RDRP group (n=77)^a^				Non-RDRP group (n=143)^b^	
	All	Narrow subgroup (n=51)^c^	Broad subgroup (n=26)^d^	p		p
Escalation, % (n)^e^	27.2% (21)	35.2% (18)	11.5% (3)	0.032*	16.8% (24)	0.080
Duration of antimicrobial therapy, d	9.0±3.6	8.8±3.3	9.5±4.3	0.378	8.8±3.8	0.603
All deaths in hospital	23.4% (18)	17.6% (9)	34.6% (9)	0.096	15.4% (22)	0.148
Death from NHCAP	13.0% (10)	11.8% (6)	15.4% (4)	0.655	4.9% (7)	0.032*
Time to death, d (median, IQR)^f^	7 (5.3–8.6)	8 (6.3–9.9)	6 (4.5–7.3)	0.170	5 (3–7)	0.684
Improved NHCAP, n (n=204,203)					
Death after improved NHCAP	11.9% (8/67)	6.7% (3/45)	22.7% (5/22)	0.057	10.911.0% (15/ 137 136)	0.8330.818

^a^ Patients with risk factors for drug-resistant pathogen infection. ^b^ Patients without risk factors for drug-resistant pathogen infection. ^c^ Patients treated with narrow-spectrum antimicrobials for NHCAP. ^d^ Patients treated with broad-spectrum antimicrobials for NHCAP. ^e^ Changed from an empirically selected antimicrobial agent to an antimicrobial agent with a broader spectrum. ^f^ Number of days between hospitalization and death from NHCAP. * Significant difference. IQR, interquartile range; NHCAP, nursing and healthcare-associated pneumonia.

**Table3:** Comparison of successful and failed narrow-spectrum antimicrobial therapy in the RDRP group^a^

	Treatment success (n=33)^b^	Treatment failure (n=18)^c^	p
Age (y)	83.4±15.0	82.3±7.8	0.782
Sex (male)	36% (12)	61% (11)	0.141
Underlying disease			
COPD	18% (6)	11% (2)	0.696
Chronic heart failure	39% (13)	33% (6)	0.767
Chronic renal failure	15% (5)	6% (1)	0.405
Diabetes	9% (3)	28% (5)	0.112
Cerebrovascular disease	39% (13)	44% (8)	0.772
Parkinson disease	15% (5)	22% (4)	0.703
Dementia	58% (19)	28% (5)	0.076
Cancer	12% (4)	0% (0)	0.284
A-DROP score ≥3	45% (15)	50% (9)	0.778
Older age^d^	84% (28)	89% (16)	1.000
BUN ≥21 mg/dL	39% (13)	44% (8)	0.772
Respiratory failure^e^	33% (11)	61% (11)	0.078
Orientation disturbance^f^	58% (19)	56% (10)	1.00
Low blood pressure^g^	6% (2)	11% (2)	0.607
Systolic blood pressure (mmHg)	132.27±24.56	119.5±30.43	0.109
Diastolic blood pressure (mmHg)	75.81±21.66	67.17±20.45	0.170
Heart rate (bpm)	92.75±23.94	92.83±20.70	0.991
WBC (/μL)	11051.5±4677.7	9511.1±4654.0	0.265
CRP (mg/dL)	9.56±8.14	11.23±10.20	0.524
BUN (mg/dL)	25.21±19.13	22.56±14.24	0.608
Hb (g/dL)	11.51±1.89	11.50±2.44	0.984
Ht (%)	35.68±5.23	34.59±7.18	0.535
Platelets (×10^4^/μL)	240.21±85.66	275.44±98.84	0.190
Alb (g/dL)	3.10±0.71	2.79±0.61	0.125
Na (mEq/L)	138.03±6.93	136.28±6.85	0.390
Transported by ambulance	30% (10)	56% (10)	0.132
Sputum culture results			
*Pseudomonas aeruginosa*	6% (2)	22% (4)	0.168
MRSA	9% (3)	11% (2)	1.00

^a^ Patients with risk factors for drug-resistant pathogen infection ^b^ Patients with initial empirical antimicrobial selection who had improved NHCAP without the need for escalation. ^c^ Patients with initial empirical antimicrobial selection who had improved NHCAP with escalation or who died. ^d^ ≥70 years for males and ≥75 years for females. ^e^ SaO_2_ ≤90% or PaO_2_ ≤60 mmHg. ^f^ Glasgow Coma Scale score <15. ^g^ Systolic blood pressure ≤90 mmHg. Escalation was defined as a change from an empirically selected antimicrobial agent to an antimicrobial agent with a broader spectrum. Escalation was performed in all six fatal cases. Alb, albumin; BUN, blood urea nitrogen; COPD, chronic obstructive pulmonary disease; CRP, C-reactive protein; Hb, hemoglobin; Ht, hematocrit; MRSA, methicillin‐resistant *Staphylococcus aureus*; NHCAP, nursing and healthcare-associated pneumonia; WBC, white blood cell.

**Table4:** Differences in underlying disease at discharge and all-cause in-hospital death

	All NHCAP patients (n=220)		
	Discharge (n=180)^a^	In-hospital death (n=40)^b^	p
Age (y)	85.1±9.5	82.2±8.2	0.073
Sex (male)	58% (104)	50% (20)	0.384
Comorbidities			
COPD	13% (24)	18% (7)	0.461
Chronic heart failure	34% (62)	38% (15)	0.717
Chronic renal failure	17% (31)	23% (9)	0.496
Diabetes	16% (29)	8% (3)	0.217
Cerebrovascular disease	31% (55)	38% (15)	0.454
Parkinson disease	10% (18)	15% (6)	0.400
Dementia	53% (96)	43% (17)	0.226
Cancer	7% (12)	5% (2)	1.000

^a^ Patients admitted with NHCAP and discharged alive. ^b^ Patients who died in hospital, including deaths from NHCAP and deaths after NHCAP improvement. COPD, chronic obstructive pulmonary disease; NHCAP, nursing and healthcare-associated pneumonia.

## References

[1] American Thoracic Society. Guidelines for the management of adults with hospital-acquired, ventilator-associated, and healthcare-associated pneumonia. Am J Respir Crit Care Med 2005; 171: 388–416. 15699079 10.1164/rccm.200405-644ST

[2] Carratalà J, Mykietiuk A, Fernández-Sabé N, Suárez C, Dorca J, Verdaguer R, Manresa F, Gudiol F. Health care-associated pneumonia requiring hospital admission: epidemiology, antibiotic therapy, and clinical outcomes. Arch Intern Med 2007; 167: 1393–1399. 17620533 10.1001/archinte.167.13.1393

[3] Park HK, Song J, Um S, Koh W, Suh GY, Chung MP, Kim H, Kwon OJ, Jeon K. Clinical characteristics of health care-associated pneumonia in a Korean teaching hospital. Respir Med 2010; 104: 1729–1735. 20605087 10.1016/j.rmed.2010.06.009

[4] Zilberberg MD, Shorr AF, Micek ST, Mody SH, Kollef MH. Antimicrobial therapy escalation and hospital mortality among patients with health-care-associated pneumonia: a single-center experience. Chest 2008; 134: 963–968. 18641103 10.1378/chest.08-0842

[5] Venditti M, Falcone M, Corrao S, Licata G, Serra P. Outcomes of patients hospitalized with community-acquired, health care- associated, and hospital-acquired pneumonia. Ann Intern Med 2009; 150: 19–26. 19124816 10.7326/0003-4819-150-1-200901060-00005

[6] Kohno S, Imamura Y, Shindo Y, Seki M, Ishida T, Teramoto S, Kadota J, Tomono K, Watanabe A. Clinical practice guidelines for nursing- and healthcare-associated pneumonia (NHCAP)[complete translation]. Respir Investig 2013; 51: 103–126. 10.1016/j.resinv.2012.11.001 23790739

[7] Koizumi T. Clinical evaluation of the outcomes in nursing and healthcare associated pneumonia in rural hospital in the high-aging area. Niigata Medical Journal 2017; 131: 147–159 (in Japanese).

[8] Nakatasuka Y, Morimoto C, Yasuda I, Tsuji T, Kaji Y, Yasuda T, Hashimoto S, Hee HM, Hajiro T, Tanaka E, Taguchi Y. An analysis of the correlation between guidelines-concordant treatment and the treatment outcome on nursing and healthcare-associated pneumonia patients. The Journal of the Japanese Association for Infectious Disease 2013; 87: 739–745 (in Japanese). 10.11150/kansenshogakuzasshi.87.739 24483021

[9] Komiya K, Ishii H, Umeki K, Muzunoe S, Okada F, Johkoh T, Kadota J. Impact of aspiration pneumonia in patients with community-acquired pneumonia and healthcare-associated pneumonia: a multicenter retrospective cohort study. Respirology 2013; 18: 514–521. 23231701 10.1111/resp.12029

[10] Grenier C, Pépin J, Nault V, Howson J, Fournier X, Poirier MS, Cabana J, Craig C, Beaudoin M, Valiquette L. Impact of guideline consistent therapy on outcome of patients with healthcare-associated and community-acquired pneumonia. J Antimicrob Chemother 2011; 66: 1617–1624. 21586592 10.1093/jac/dkr176

[11] Kett DH, Cano E, Quartin AA, Mangino JE, Zervos MJ, Peyrani P, Cely CM, Ford KD, Scerpella EG, Ramirez JA. Implementation of guidelines for management of possible multidrug-resistant pneumonia in intensive care: an observational, multicenter cohort study. Lancet Infect Dis 2011; 11: 181–189. 21256086 10.1016/S1473-3099(10)70314-5

